# First 2 Months of Operation at First Publicly Recognized Overdose Prevention Centers in US

**DOI:** 10.1001/jamanetworkopen.2022.22149

**Published:** 2022-07-15

**Authors:** Alex Harocopos, Brent E. Gibson, Nilova Saha, Michael T. McRae, Kailin See, Sam Rivera, Dave A. Chokshi

**Affiliations:** 1 New York City Department of Health and Mental Hygiene, Queens, New York; 2 OnPoint NYC, New York, New York

## Abstract

This quality improvement study investigates outcomes and services used during the first 2 months of operation at the first 2 publicly recognized US overdose prevention centers.

## Introduction

In New York City (NYC), there were 2062 overdose fatalities in 2020, the deadliest year on record for NYC and the US.^1^ Fentanyl and its analogs were the most common substances involved in overdose deaths in NYC, present in 77% of such deaths in 2020.^1^ A characteristic of fentanyl-involved overdose is rapid onset of overdose symptoms^2^; however, with timely administration of oxygen or naloxone, deaths can be averted.

In response to unprecedented numbers of overdose deaths, on November 30, 2021, NYC implemented overdose prevention center (OPC) services at 2 syringe service programs operated by OnPoint NYC. Also known as supervised consumption sites, OPCs are health care facilities that aim to improve individual and community health, increase public safety, and reduce consequences of drug use, including overdose deaths, public drug use, and syringe litter.^3,4^ Operating in more than 10 countries, OPCs offer supervised, hygienic spaces in which people can use preobtained drugs and access services, onsite or by referral, to health and mental health care, drug treatment, and other social supports.^3,4^ While previous research documented operations at an underground US OPC,^5^ use of sanctioned sites has not yet been studied, to our knowledge. This study describes the first 2 months of operation and use at the first 2 publicly recognized US OPCs.

## Methods

Because data were collected for program evaluation and presented in aggregate, the NYC Department of Health and Mental Hygiene Institutional Review Board (IRB) determined that this quality improvement study was not human participants research and so IRB approval and informed consent were not required. This study is reported following the SQUIRE reporting guideline.

Data were collected by program staff from individuals using services at 2 OPC sites at intake and before each subsequent use of OPC services. Outcome data related to OPC visits (eg, staff interventions to mitigate overdose risk) were also recorded. Using a unique identifier, OPC participant data were then matched with data indicating uptake of additional services provided at the syringe service program. Descriptive statistics (frequencies and percentages) were calculated using SAS statistical software version 9.4 (SAS Institute).

## Results

Between November 30, 2021, and January 31, 2022, 613 individuals used OPC services 5975 times across 2 sites. Most individuals identified as male (78.0%), and 55.3% identified as Hispanic, Latino, or Latina. The mean (range) age was 42.5 (18-71) years. A plurality of individuals (36.9%) reported being street homeless. Fewer than one-fifth of individuals (17.8%) were living in their own rooms or apartments (Table).

**Table.** Demographic Characteristics by Enrollment Site^a^

Characteristic	Participants, No. (%)		
	Total (N = 613)	East Harlem (n = 405)	Washington Heights (n = 208)
Gender identity			
Man	478 (78.0)	326 (80.5)	152 (73.1)
Woman	123 (20.1)	72 (17.8)	51 (24.5)
Transgender woman	10 (1.6)	5 (1.2)	5 (2.4)
Nonbinary or GNC	2 (0.3)	2 (0.5)	0
Race and ethnicity^b^			
Hispanic, Latino, or Latina	339 (55.3)	223 (55.1)	116 (55.8)
Non-Hispanic			
Black	109 (17.8)	92 (22.7)	17 (8.2)
White	146 (23.8)	79 (19.5)	67 (32.2)
Other race	14 (2.3)	8 (2.0)	6 (2.9)
Declined to answer or missing	5 (0.8)	3 (0.7)	2 (1.0)
Age group, y			
18-29	59 (9.6)	25 (6.2)	34 (16.3)
30-39	187 (30.5)	110 (27.2)	77 (37.0)
40-49	207 (33.8)	144 (35.6)	63 (30.3)
50-59	122 (19.9)	93 (23.0)	29 (13.9)
≥60	38 (6.2)	33 (8.1)	5 (2.4)
Housing status			
Homeless on street	226 (36.9)	123 (30.4)	103 (49.5)
Renting apartment	106 (17.3)	71 (17.5)	35 (16.8)
Shelter or SRO	108 (17.6)	81 (20.0)	27 (13.0)
Staying with family or friends	62 (10.1)	33 (8.1)	29 (13.9)
Own home	3 (0.5)	1 (0.2)	2 (1.0)
Declined to answer or missing	108 (17.6)	96 (23.7)	12 (5.8)

Abbreviations: GNC, gender nonconforming; SRO, single room occupancy.

^a^ Data source was OnPoint NYC overdose prevention center enrollment, November 30, 2021, to January 31, 2022.

^b^ Race and ethnicity were self-identified and were collected because of a commitment to measuring reach and access of overdose response programs to assess potential disparities in care. Original survey categories for race and ethnicity were Asian, Black, Hawaiian or Pacific Islander, Hispanic or Latino, Native American or Alaskan Native, and White. Latino or Latina includes individuals of Hispanic origin based on ancestry reported at enrollment, regardless of reported race. Black and White race categories do not include individuals of Latino or Latina origin. Non-Hispanic was added to Black and White categories because individuals who identified as Hispanic, Latino, or Latina were not included in these groups. Native American or Alaska Native and Native Hawaiian or Pacific Islander groups were collapsed because numbers were small. Other race includes Asian, Middle Eastern or North African (write-in responses), Native American or Alaska Native, and Native Hawaiian or Pacific Islander.

In self-reported data, the drug most commonly used across 2 sites was heroin or fentanyl (73.7%) and the most frequent route of drug administration at the OPC was injection (65.0%). Among all participants, 75.9% reported that they would have used their drugs in a public or semipublic location if OPC services had not been available (Figure).

**Figure.**
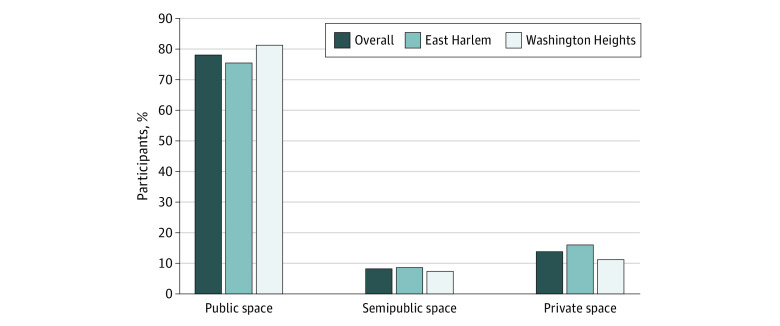
Reported Drug Use Location If Overdose Prevention Center Was Not an Option Data source was OnPoint overdose prevention center enrollment, November 30, 2021, to January 31, 2022. Additional categories not displayed in this figure are other (0.6%) and missing or decline to answer (2.5%). Private space indicates own residence or other’s residence; public space, street, sidewalk, underpass, park, or between cars; semipublic space, public bathroom, subway station, syringe service program bathroom, hotel, shelter, or building roof, hallway, or basement.

During the first 2 months of OPC operation, trained staff responded 125 times to mitigate overdose risk. In response to opioid-involved symptoms of overdose, naloxone was administered 19 times and oxygen 35 times, while respiration or blood oxygen levels were monitored 26 times. In response to stimulant-involved symptoms of overdose (also known as overamping), staff intervened 45 times to provide hydration, cooling, and de-escalation as needed. Emergency medical services responded 5 times, and participants were transported to emergency departments 3 times. No fatal overdoses occurred in OPCs or among individuals transported to hospitals.

More than half of individuals using OPC services (52.5%) received additional support during their visit. This included, but was not limited to naloxone distribution, counseling, hepatitis C testing, medical care, and holistic services (eg, auricular acupuncture).

## Discussion

This quality improvement study found that during the first 2 months of operations, services at 2 OPCs in NYC were heavily used, with early data suggesting that supervised consumption in these settings was associated with decreased overdose risk. Data also suggested that OPCs were associated with decreased prevalence of public drug use. Findings are limited by the short study period and lack of a comparison group with individuals not participating in OPC services. Additional evaluation may explore whether OPC services are associated with improved overall health outcomes for participants, as well as neighborhood-level outcomes, including public drug use, improperly discarded syringes, and drug-related crime.
